# Effect of Hepatitis C Virus Genotype, Cirrhosis, and Viral Cure on Serum Phosphatidylinositol Species Profiles

**DOI:** 10.3390/biomedicines13112720

**Published:** 2025-11-06

**Authors:** Kilian Weigand, Georg Peschel, Marcus Höring, Sabrina Krautbauer, Gerhard Liebisch, Martina Müller, Christa Buechler

**Affiliations:** 1 Department of Internal Medicine I, Gastroenterology, Hepatology, Endocrinology, Rheumatology, and Infectious Diseases, University Hospital Regensburg, 93053 Regensburg, Germany; kilian.weigand@gk.de (K.W.); georg.peschel@klinikum-ffb.de (G.P.); martina.mueller-schilling@klinik.uni-regensburg.de (M.M.); 2 Department of Gastroenterology, Gemeinschaftsklinikum Mittelrhein, 56073 Koblenz, Germany; 3 Department of Internal Medicine, Klinikum Fürstenfeldbruck, 82256 Fürstenfeldbruck, Germany; 4 Institute of Clinical Chemistry and Laboratory Medicine, University Hospital Regensburg, 93053 Regensburg, Germany; marcus.hoering@klinik.uni-regensbug.de (M.H.); sabrina.krautbauer@klinik.uni-regensburg.de (S.K.); gerhard.liebisch@klinik.uni-regensbug.de (G.L.)

**Keywords:** liver cirrhosis, lipid species, genotype, hepatitis C

## Abstract

**Background/Objectives:** Phosphatidylinositol (PI) species are bioactive lipids implicated in liver fibrogenesis. Hepatitis C virus (HCV) relies on host lipid metabolism for infection. The relationship between serum PI profiles, chronic HCV, and liver injury remains incompletely defined. **Methods:** Fourteen PI species were quantified by direct flow injection–tandem mass spectrometry (FIA–MS/MS; triple quadrupole) in serum from 178 patients with chronic HCV at three time points: before treatment and at weeks 4 and 12 after starting direct-acting antiviral (DAA) therapy. **Results:** At baseline, PI 34:1, 36:1, and 36:3 were higher in patients with ultrasound-diagnosed cirrhosis than in those without, whereas PI 38:4, 40:5, and 40:6 were lower. In non-cirrhotic patients, PI 36:3, 36:4, 38:3, 38:4, and 38:5 increased, while PI 40:5 and 40:6 declined at weeks 4 and 12 after therapy start. In cirrhosis, viral cure was not associated with changes in PI species. By the end of therapy, cirrhotic patients showed higher PI 36:3 and lower PI 38:4 than non-cirrhotic patients. Genotype 3a was associated with lower PI 38:3, 38:4, and 38:5; the reduction in PI 38:4 persisted to the end of therapy. Across time points, most PI species did not correlate with routine markers of liver injury or inflammation. **Conclusions:** HCV cure remodels the serum PI profile in non-cirrhotic patients. These findings suggest that altered PI profiles are primarily linked to HCV infection, supporting a role for PI lipids in viral propagation.

## 1. Introduction

Chronic hepatitis C virus (HCV) infection increases systemic and hepatic inflammation, the latter of which contributes to the development and progression of liver fibrosis, which can ultimately result in liver cirrhosis [1]. Direct-acting antivirals (DAAs) can effectively inhibit viral replication and cure HCV within weeks, achieving a sustained virological response (SVR) of up to 100% [2]. Eliminating HCV reduces inflammation and the rate of liver cirrhosis complications and mortality [3,4]. However, whether fibrosis progression is halted or even resolved remains an open question [3,5].

Host lipid metabolites are required for HCV propagation, and the low-density lipoprotein (LDL) levels of patients with chronic HCV infection are lower than those of healthy controls [6,7,8,9,10,11]. Recovery from HCV normalizes serum cholesterol and LDL levels [8,9,10,11]. LDL is composed of different lipid classes, and lysophosphatidylcholine levels in serum increase in parallel with LDL at four weeks post-initiation of DAA therapy [12,13]. Phosphatidylinositol (PI) is a less abundant lipid class that contributes 2–10% of phospholipids in human serum [14,15]. Mammalian PIs have a characteristic fatty acid profile with C18:0 enriched at the sn-1 and C20:4 at the sn-2 position, with PI 38:4 being the predominant species [14]. Phosphorylated forms of PI are of great interest, as they have many different functions, such as cell signaling via phosphoinositide 3-kinases [14]. Moreover, PI enhances cellular cholesterol export to high-density lipoprotein (HDL) and hepatic cholesterol clearance, which can protect against cardiovascular disease [16,17]. Patients with chronic HCV infection are at higher risk of cardiovascular disease [18], but to our knowledge, serum levels of PI species have not been studied in HCV patients before or after viral cure.

The association of PIs with liver fibrosis is supported by studies showing that the TT genotype of the rs641738 C > T polymorphism in the membrane-bound O-acyltransferase domain-containing 7 (MBOAT7) gene is associated with fibrosis in patients with metabolic dysfunction-associated steatotic liver disease (MASLD) [19,20]. The MBOAT7 rs641738 C > T polymorphism has also been recognised as a predisposing locus for cirrhosis in alcoholic liver disease [21]. MBOAT7 is highly expressed in immune cells, but it is also expressed in hepatocytes and encodes a lysophosphatidylinositol acyltransferase 1 with a specificity for C20:4 coenzyme A at the sn-2 position of PIs [14,22]. The rs641738 T allele is associated with reduced hepatic expression and thus lower enzymatic activity of MBOAT7, consistent with reduced plasma concentrations of PI 36:4, PI 38:3, PI 38:4, and PI 38:5 relative to total PI levels, while concentrations of most other PI species are increased [23].

The TT genotype of MBOAT7 has also been associated with advanced fibrosis in patients with HCV [20]. A study of 2,051 HCV patients found that the rs641738 T variant was associated with severe hepatic inflammation, an increased risk of fibrosis, and rapid fibrosis progression [24]. However, a subsequent study failed to identify an association between the rs641738 C > T polymorphism and fibrosis progression in HCV patients [25].

The PI species in the blood of patients with chronic liver disease have rarely been analyzed. One study showed normal levels of the PI species with 18:2n6, 18:3n3, 20:4n6, and 20:5n3 acyl chains in the plasma of patients with non-alcoholic steatohepatitis, whereas PIs with 22:6n3 fatty acid were increased in patients with MASLD [26]. Total PI levels in the blood of patients with MASLD have been found to be either normal or increased [26,27]. It is not yet clear whether distinct PI species are associated with liver disease severity.

Liver cirrhosis is the final stage of progressive liver disease, irrespective of the cause, and the serum lipid and lipoprotein levels of patients with this condition are usually low [28,29]. However, plasma levels of PI 35:2 and PI 37:2 were increased in patients with liver cirrhosis compared with healthy controls [30], and higher PI levels in patients with liver cirrhosis have been reported before [31].

Our study aimed to assess the serum levels of 14 PI species in patients with HCV, both before and at the end of DAA therapy, to examine the effect of chronic HCV infection on circulating PI levels, and to identify potential associations with viral load, viral genotype, and the severity of liver disease. Given the association of the MBOAT7 polymorphism with liver injury in HCV [20], a PI profile consistent with reduced activity of this enzyme was anticipated. Furthermore, since most analyzed lipids decline after viral cure [11], it was suggested that this would also occur with PIs.

## 2. Materials and Methods

### 2.1. Study Cohort

Sera from patients who had not previously received treatment for HCV infection were collected at the Department of Internal Medicine I at the University Hospital of Regensburg between October 2014 and September 2019. It is important to note that the patients’ blood was not collected after fasting and that non-fasting serum was used for lipid analysis.

All patients were deemed eligible for DAA therapy in accordance with the European Association for the Study of the Liver’s guidelines [2]. Participants had to be over 18 years of age. Exclusion criteria included co-infection with the human immunodeficiency virus (HIV) or the hepatitis B virus (HBV). Patients with decompensated liver cirrhosis were also excluded.

Patients diagnosed with chronic HCV received treatment with one of the following regimens: sofosbuvir/velpatasvir, glecaprevir/pibrentasvir, sofosbuvir/daclatasvir, sofosbuvir/ledipasvir or elbasvir/grazoprevir. This was in accordance with international guidelines [2]. Most patients were enrolled shortly after the approval of interferon-free direct-acting antiviral (DAA) therapies, during a period when the adverse effects of statins were anticipated [32]; consequently, the use of statins was suspended during therapy.

Liver cirrhosis was diagnosed via ultrasound based on the presence of a nodular liver surface, reduced liver size, and coarse liver parenchyma [33]. We also performed Acoustic Radiation Force Impulse for non-invasive measurement of liver stiffness. The cut-offs used to define fibrosis for the fibrosis-4 (FIB-4) score were as follows: Fibrosis: >3.25, No fibrosis: <1.3 for patients under 65 years old and <2 for patients over 65 years [34]. Analysis of laboratory parameters was conducted at the Institute of Clinical Chemistry and Laboratory Medicine at the University Hospital Regensburg.

The 114 healthy controls (47 females) had a median age of 50 (20–93) years. All controls were in good health and maintained a normal body mass index (BMI).

### 2.2. The Measurement of PI Species

Lipids were isolated from 10 µL of serum in accordance with the extraction protocol described by Bligh and Dyer [35]. For quantification, non-naturally occurring PI 15:0/18:1[D7] (Avanti Polar Lipids, Alabaster, AL, USA) was incorporated prior to lipid extraction. A total volume of 2 mL of chloroform was employed for the extraction. Following this, 1 mL of the chloroform phase was isolated, vacuum-dried, and subsequently dissolved in a methanol (Merck, Darmstadt, Germany)/chloroform (Roth, Karlsruhe, Germany) mixture at a ratio of 3:1 (*v*/*v*), which contained 7.5 mM ammonium acetate [36]. Lipid analysis was conducted using direct flow injection analysis (FIA) coupled with a triple quadrupole mass spectrometer (FIA-MS/MS, QQQ) operated in positive ion mode using the setup described previously [36]. A neutral loss of 277 was used to quantify PI species [37]. Data analysis involved correction of Type-II isotopic overlap [36] and Type-I isotopic effects [38]. Quantification was achieved based on the amount of the internal standard. Lipid species were classified according to the recently published guidelines for shorthand notation of lipid structures derived from mass spectrometry [39]. Further details of the workflow can be found in the lipidomics checklist [38], which is available as Supplementary Data (Table S1).

### 2.3. Statistical Analysis

Normality was assessed with the Shapiro–Wilk test; only PI 38:4 and PI 40:5 were normally distributed, and non-parametric tests were used for analysis of all PI species. Accordingly, PI species are presented as box plots and summarized as medians with ranges (minimum–maximum). Between-group comparisons used the Mann–Whitney U test; multi-group comparisons used the Kruskal–Wallis test; and diagnostic discrimination was assessed by receiver operating characteristic curve (ROC) analysis. Paired groups were compared using a Student’s *t*-test (Ms Excel). All other analyses were performed using IBM SPSS Statistics, version 26 (IBM Corp., Armonk, NY, USA). All *p*-values were adjusted for multiple comparisons by multiplying them by 14, the number of different PI species analyzed. A two-sided *p*-value of less than 0.05 was considered statistically significant.

## 3. Results

### 3.1. PI Species of Patients with and Without Liver Cirrhosis

The study included 178 patients, 40 of whom were diagnosed with liver cirrhosis by ultrasound. Previous studies have shown that patients with liver cirrhosis have higher levels of PI [30,31]. Indeed, the PI levels of patients with HCV and cirrhosis were increased for PI 34:1, 36:1, and 36:3 (Table 1). However, PIs 38:4, 40:5, and 40:6 were significantly reduced in patients with cirrhosis (Table 1). Total PI levels in HCV infected patients with and without cirrhosis were similar (Table 1).

As liver cirrhosis was associated with altered PI species levels, these 40 patients were excluded from further analysis. Details of patients with chronic HCV without cirrhosis are summarized in Table 2.

### 3.2. Correlation of PI Species with Laboratory Measures of Liver Function and Markers of Systemic Inflammation

The PI species did not correlate with bilirubin, the international normalized ratio, or creatinine, and were accordingly not related to the MELD score (see Table 3). There were no significant associations with albumin or alanine aminotransferase (ALT). PIs 38:6, 40:5, and 40:6 positively correlated with aspartate aminotransferase (AST). PI 36:2 positively correlated with HDL and PIs 38:4 and 40:4 with LDL (Table 3). PI species were not associated with C-reactive protein or procalcitonin (*p* > 0.05 for all). A negative association was observed between PI 38:5 and leukocyte number.

### 3.3. Correlation of PI Species with Viral Load and Associations with Viral Genotype

**Figure 1:**
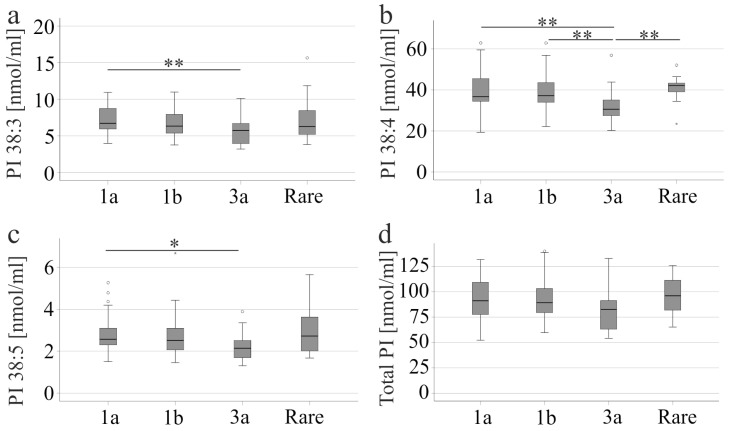
PI levels in the serum of patients with different HCV genotypes. (**a**) PI 38:3; (**b**) PI 38:4; (**c**) PI 38:5, and (**d**) total PI levels of patients infected with genotype 1a, 1b, 3a or rare variants, which are grouped together. * *p* < 0.05, ** *p* < 0.01. The figure shows outliers as small circles and asterisks.

Of the patients without liver cirrhosis, 43 were infected with genotype 1a, 53 with genotype 1b, 29 with genotype 3a and 13 with less common HCV genotypes, which were grouped together. PI 38:3 and 38:5 were lower in genotype 3a patients than in genotype 1a patients. PI 38:4 was lowest in genotype 3a patients compared to all other groups (Figure 1a–c). PI species levels/total PI levels were similar between the different genotypes (*p* > 0.05) and only PI 36:1/total PI level was modestly higher in genotype 3a compared to 1b (*p* = 0.017). Total PI level did not significantly change in genotype 3a patients compared to patients infected with different genotypes (Figure 1d).

The viral load of patients infected with genotype 3a did not differ from the other groups. CRP, procalcitonin, leukocyte number, thrombocytes, HDL, LDL, bilirubin, albumin, creatinine, MELD score, and AST of genotype 3 infection were similar to other genotypes (*p* > 0.05 for all). These patients had higher ALT (*p* < 0.001) than patients with rare genotypes.

Liver steatosis was observed in 37% of patients with genotype 1a, 34% with genotype 1b, 48% with genotype 3a, and 38% with rare genotypes. There was no difference between these groups (*p* > 0.05). It should be noted that the PI profiles of patients with and without liver steatosis were similar (*p* > 0.05).

Sofosbuvir/ledipasvir was not recommended for treatment of patients with genotype 3a [2], and was used in 24, 13, 0, and 2 patients with genotype 1a, 1b, 3a and rare genotypes, respectively (*p* < 0.001).

Serum PI species levels did not correlate with viral load (*p* > 0.05 for all).

### 3.4. PI Species Post-DAA Therapy

**Figure 2:**
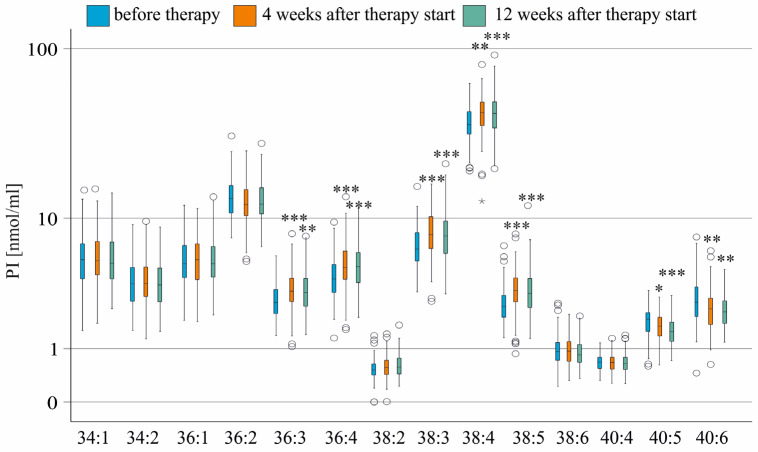
PI species in the serum of patients without liver cirrhosis during therapy. PI species levels before (blue boxes), at 4 weeks (orange boxes), and 12 weeks (green boxes) after the start of therapy are shown. * *p* < 0.05, ** *p* < 0.01 and *** *p* < 0.001 compared to levels before treatment. The figure shows outliers as small circles and asterisks.

At 12 weeks after DAA therapy, patients had lower levels of ferritin, ALT, AST, and procalcitonin. LDL was significantly increased (Table 2) in accordance with previous reports [11,40,41,42,43]. Four and 12 weeks after therapy initiation, PI 36:3, 36:4, 38:3, 38:4, and 38:5 increased significantly. PI 40:5 and 40:6 declined (Figure 2). Levels of these PI species did not further change from 4 to 12 weeks after therapy started (Figure 2).

During HCV cure, the percentage of PI 36:2, 38:5, 40:5, and 40:6 declined, while the percentage of PI 36:4, 38:3, and 40:4 increased, with the percentage of PI 38:4 remaining unchanged (Figure S1).

In patients with cirrhosis, the levels of PI species before therapy and at the end of therapy were similar (*p* > 0.05 for all). This may be explained by the lower number of patients in this cohort. However, the % change in the median PI levels was also lower in cirrhosis (Table 4). It should be noted that the viral titer of patients without cirrhosis was 1150 (0–25,000) × 10^3^ U/mL, and it was 470 (45–6500) × 10^3^ U/mL in those with cirrhosis (*p* = 0.046).

To ensure that the exclusion of patients with liver cirrhosis did not introduce a confounding factor, the PI levels of the entire cohort (including patients with liver cirrhosis) were analysed during therapy. The details of this cohort can be found in a recent paper published by our group [44]. In the entire cohort, PI 36:3, 36:4, 38:3, and 38:5 were higher than before treatment (*p* < 0.001 for all), with similar levels at four and 12 weeks after the start of therapy. Levels of PI 40:6 were significantly decreased at four and 12 weeks after the start of therapy (*p* < 0.01). PI 40:5 levels were lower at the end of therapy than before treatment (*p* < 0.05). These results demonstrate that changes in the PI profile are comparable in the entire cohort and in the subgroup of patients without liver cirrhosis. During HCV cure, in the entire cohort the percentage of PI 36:2 (*p* < 0.001), 40:4 (*p* < 0.01), 40:5 (*p* < 0.001), and 40:6 (*p* < 0.001) declined, while the percentage of 36:4 (*p* < 0.001), 38:3 (*p* < 0.001), and 38:5 (*p* < 0.001) increased, with the percentage of PI 38:4 remaining unchanged.

The percentage changes in PI 36:2, 40:5, 40:6, 36:4, and 38:3 were consistent across the entire cohort and the subgroup from which patients with liver cirrhosis had been excluded. The main PI species did not change in any case (PI 38:4).

At the end of therapy, there was no correlation between PI species and the MELD score, bilirubin, INR, creatinine, ALT, and AST. PI species were not associated with C-reactive protein, procalcitonin, or leukocyte count. PI 36:4, 38:3, 38:4, 40:4, and 40:5 positively correlated with LDL, and none of the PI species correlated with HDL (Table 5).

The PI 38:5 level in patients with genotype 3a was lower than in patients infected with genotypes 1a (*p* = 0.039) or 1b (*p* = 0.006). However, the genotype-related differences in PI 38:3 and 38:4 described above disappeared.

### 3.5. PI Species of Patients with and Without Cirrhosis Post-DAA Therapy

By the end of the therapy, the PI 36:3 levels were higher in patients with liver cirrhosis (*p* = 0.012), while the PI 38:4 levels were lower (*p* < 0.001) in comparison to patients without liver cirrhosis. However, PI 36:3 and PI 38:4 did not discriminate patients with and without liver cirrhosis defined by ultrasound (area under the receiving operating characteristic curve (AUROC) were 0.213 ± 0.040 and 0.672 ± 0.049, respectively).

The Fibrosis-4 (FIB-4) score was also employed to define patients with liver cirrhosis. The FIB-4 index can correctly discriminate patients with HCV who have no liver fibrosis and those with advanced fibrosis. However, it cannot accurately diagnose intermediate fibrosis stages [45]. Of the patients in our cohort, 109 did not have fibrosis, while 31 did have advanced fibrosis. Patients with fibrosis had higher serum levels of PI 34:1, 34:2, 36:1, 36:3 and 38:2, and lower levels of PI 38:4 (Table S2). Changes to PI 36:3 and 38:4 were consistent when cirrhosis was diagnosed using ultrasound or the FIB-4 score.

The AUROC for discriminating between patients with a high FIB-4 score and those with a low or intermediate score was 0.714 ± 0.048 for PI 36:3 and 0.276 ± 0.051 for PI 38:4. The AUROC of the MELD score was 0.877 ± 0.032 following DAA treatment, indicating its ability to differentiate between cirrhotic and non-cirrhotic patients with high accuracy.

### 3.6. Comparison of HCV Patients Post-DAA Therapy and Controls

**Figure 3:**
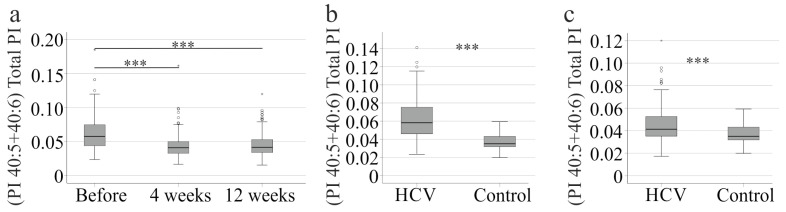
PI 40:5 and 40:6 to the total PI level. (**a**) (PI 40:5 + 40:6) to total PI level of HCV patients before therapy, at 4 and 12 weeks after therapy start. (**b**) (PI 40:5 + 40:6) to total PI level of patients with HCV before therapy in comparison to 114 healthy controls; (**c**) (PI 40:5 + 40:6) to total PI level of patients with HCV without liver cirrhosis at therapy end in comparison to 114 healthy controls. *** *p* < 0.001. The figure shows outliers as small circles and asterisks.

The ratio of PI 40:5 + 40:6 to total PI decreased four weeks after therapy commenced and remained unchanged by the end of therapy (Figure 3a). This suggests that the PI profile normalises during viral clearance. Currently, absolute lipid levels that are not measured simultaneously cannot be compared due to variations in different analyses. Healthy controls were also not included in the current study. However, lipid ratios of different analyses should be similar. Therefore, the (PI 40:5 + 40:6)/total PI ratio was compared between patients with HCV and 114 healthy controls and was found to be higher in the patients before therapy (Figure 3b). By the end of the therapy, the ratio of PI (40:5 + 40:6) to total amount of PI in patients with HCV was still higher than in healthy controls, indicating that the levels of these species had not normalised completely (Figure 3c).

### 3.7. PI Species in Relation to Age, Sex, and Body Mass Index

Age, sex, and body mass index may be confounding factors when analyzing serum lipids. PI 38:6 (r = 0.264, *p* = 0.024), PI 40:5 (r = 0.305, *p* = 0.004), and PI 40:6 (r = 0.312, *p* = 0.003) were found to correlate with age at the start of therapy in patients without cirrhosis. At the end of therapy, PI 34:1 (r = 0.272, *p* = 0.019), PI 34:2 (r = 0.273, *p* = 0.019), PI 36:3 (r = 0.256, *p* = 0.037), PI 36:4 (r = 0.418, *p* < 0.001), PI 38:5 (r = 0.303, *p* = 0.005), PI 38:6 (r = 0.273, *p* = 0.018), PI 40:4 (r = 0.254, *p* = 0.040) and PI 40:5 (r = 0.306, *p* = 0.004) correlated with age. PI 36:3 was the only species that differed between the sexes, being higher in females both before therapy (*p* = 0.023) and at the end of therapy (*p* = 0.016). There was no correlation between PI species and BMI before treatment and at the end of therapy (*p* > 0.05 for all).

The BMI and sex distributions of patients with and without cirrhosis diagnosed by ultrasound were similar. However, patients with liver cirrhosis were older (*p* < 0.001), suggesting that changes in PI species 36:3 in cirrhosis may be confounded by higher age. Nevertheless, the difference in PI 36:3 between patients with and without liver cirrhosis (*p* = 0.049) was still significant after adjusting for age.

## 4. Discussion

HCV clearance after DAA therapy was associated with significant remodelling of the serum PI profile. Compared with non-cirrhotic patients, those with cirrhosis had higher PI 36:3 and lower PI 38:4. These opposing shifts suggest that individual PI species may play distinct roles in HCV infection and cirrhosis.

The diagnosis of liver cirrhosis in our patients was made using ultrasound, which is effective in identifying compensated cirrhosis in patients with chronic HCV, but has low sensitivity, meaning some cases of cirrhosis were overlooked [33,46]. A liver biopsy, considered the gold standard for diagnosing fibrosis/cirrhosis, was not performed. This is because liver biopsy cannot be done routinely due to its invasiveness. It can also produce false-negative results in up to 30% of cases, and there is a risk of adverse events [47,48].

Tests that use serological markers have been validated for the diagnosis of liver cirrhosis [48]. The FIB-4 score uses platelet count, age, AST, and ALT levels to calculate the score. As AST and ALT levels decrease rapidly after viral clearance, this score improves significantly [49]. Fibrosis will not regress within 12 weeks of therapy. Thus, chronic inflammation in HCV is related to a high FIB-4 score [49]. The AST to Platelet Ratio Index (APRI) also uses AST, which declines rapidly after viral cure [48,49]. When used in the context of HCV infection, these scores may overestimate the prevalence of cirrhosis. In the current study, the FIB-4 score predicted advanced fibrosis in 31 patients after viral cure, whereas ultrasound identified cirrhosis in 40 patients. This shows that it is still difficult to diagnose cirrhosis correctly using non-invasive tools.

Liver cirrhosis is primarily linked to low systemic lipid levels [28,50]. However, PI 36:3 is an exception, with approximately 130% higher levels observed in patients with cirrhosis defined by ultrasound or the FIB-4 score after HCV eradication. Previous analyses have reported higher levels of PI 35:2 and PI 37:2 in patients with decompensated liver cirrhosis than in healthy controls [30], but these species were not analyzed in the current study. PI 38:4 levels were reduced to approximately 70% in cirrhosis patients defined by ultrasound and to about 80% in those with a high compared to patients with a low FIB-4 score. PI 38:4 is a main product of MBOAT7 [23]; however, it is unclear whether the activity of this enzyme is reduced in liver cirrhosis. Overall, the current analysis showed that PI species do not generally change in chronic HCV patients with liver cirrhosis. Accordingly, PI species mostly did not correlate with measures of liver disease in non-cirrhosis patients before or after therapy.

Higher levels of PI 34:1 and 36:1, and lower levels of PI 40:5 and 40:6, were observed in patients with cirrhosis prior to DAA therapy. PI 40:5 and 40:6 declined during therapy in patients without cirrhosis and remained unchanged in patients with cirrhosis; consequently, the levels of patients with and without cirrhosis were similar at the end of therapy. PI 34:1 levels declined modestly during treatment (from a median of 5.4 nmol/mL to 5.0 nmol/mL), and a difference between cirrhotic and non-cirrhotic patients did not exist at the end of the study. The levels of PI 36:1 did not change during DAA therapy; however, a higher range of values may have prevented a significant difference between patients with and without cirrhosis at therapy end.

A comparison of the PI levels in non-cirrhotic patients before and after treatment showed that the levels of PIs 36:3, 36:4, 38:3, 38:4, and 38:5 increased, whereas the levels of PIs 40:5 and 40:6 decreased. This suggests that HCV propagation is related to changes in these PI species. The ratio of PI 40:5 and 40:6 to the total amount of PI in patients with HCV decreased during treatment but remained higher than in healthy controls by the end of therapy. This suggests that a cure for HCV is associated with a PI profile that is more similar to that of healthy controls, but still different. It remains to be clarified whether serum PI levels normalize during longer follow-up. The complex changes in the levels of PI species during viral clearance suggest that the HCV-specific PI profile is relevant to HCV propagation. However, distinct PI species did not correlate with viral load, suggesting that chronic HCV may be associated with the PI profile rather than specific PI species.

Phosphatidylinositol synthase lacks acyl chain selectivity, and mammalian cells can synthesise various PI species in vitro. This suggests that the altered PI profile observed in HCV infection is not due to changes in the activity of this enzyme [51]. The enzymes that modify the PI species profile are MBOAT7 and PI acyl-transferase LYCAT. However, silencing LYCAT did not alter the acyl chain composition of PIs [52]. Lower expression of MBOAT7 was associated with reduced concentrations of PIs 36:4, 38:3, 38:4 and 38:5 in plasma relative to total PI levels, while the concentrations of most other PIs are increased [23]. During HCV cure, the percentage of PI 36:2, 38:5, 40:5 and 40:6 declined, while the percentage of PI 36:4, 38:3, and 40:4 increased, with the percentage of PI 38:4 remaining unchanged, thus excluding altered activity of this enzyme as an underlying mechanism. HCV infection also inhibits the tumour suppressor p53 [53], which has been shown to shift the PI fatty acid profile towards saturated and monounsaturated variants [14]. As we did not observe a shift towards less saturated PI species, it is unlikely that p53 is involved. Currently, we cannot provide information on the underlying pathways.

The PI profile of patients with HCV and liver cirrhosis does not change significantly after the virus is eradicated. In fact, the viral load in cirrhosis patients was lower than in non-cirrhosis patients in accordance with findings from others [54]. However, viral titer did not correlate with PI species levels in our patients, indicating a minor, if any, role for PI species levels. With the exception of PI 40:5 and 40:6, a similar trend was observed for PIs 36:3, 36:4, 38:3, 38:4, and 38:5 in both cirrhotic and non-cirrhotic patients, suggesting that the smaller number of cirrhotic patients during therapy prevented these comparatively fewer changes in PI species levels during therapy from becoming significant. The less pronounced effect of DAA therapy on PI levels in patients with cirrhosis suggests an altered interaction between HCV and PI species by changes induced by the diseased liver. However, which local or systemic influence may cause this response is currently unknown.

Patients infected with genotype 3a did not differ from patients infected with genotype 1a with regard to inflammation, lipid levels, and measures of liver disease severity. PI 38:4 was found to be lower in genotype 3a patients than in all other groups. PI 38:3 and 38:5 were lower in genotype 3a patients than in genotype 1a patients. The finding that the relative abundance of all PI species, i.e., the proportion of each species to total PI levels, did not change in genotype 3 infection shows that the abundance of most PI species decreased, with this effect being significant for three species. Genotype 3 infection appears to cause a non-significant decline in PI levels in serum via an unknown pathway. Moreover, the decline in PI 38:5 persisted until the end of therapy. There is no evidence that this change contributes to the more rapid progression of liver fibrosis in those patients after viral cure [55].

Most PI species did not correlate with HDL before or at the end of therapy. Positive correlations of total PI and PIs 36:4, 38:3, 38:4, 40:4, and 40:5 with LDL were significant at 12 weeks after the start of therapy. A decline in PI species was observed in patients with familial hypercholesterolemia who responded to statin therapy [56], which is consistent with the positive correlation between PI species and LDL. The extent to which positive associations of PI species with LDL levels correspond to normal situations has not been studied in great detail.

Higher PI levels have been observed in older populations compared to younger ones [57], and at the end of therapy, most PI species were found to positively correlate with age. It should be noted that the correlations between PI species and LDL, as well as between PI species and age, are mostly significant at the end of therapy. This is consistent with the idea that HCV infection greatly affects the profile of PIs, which normalize upon elimination of the virus.

In a healthy population, PI species did not differ by sex [58], whereas in our cohort, PI 36:3 was modestly higher in females before and after therapy. Further studies are needed to evaluate whether this sex-specific difference is specific to chronic HCV. In patients with HCV, PI species did not correlate with BMI, thus excluding overweight as a confounding factor.

The limitations of this study are as follows: (1) The reproducibility of PI profile changes, e.g., in patients with genotype 3a or during therapy, was not verified using samples from other centres. (2) Serum was not collected in the fasting state. The difference in serum PI levels and/or profiles between fasting and non-fasting serum has not yet been evaluated. We cannot exclude the possibility that PI species levels differ between the fed and fasted states. (3) The serum lipids of the control group were not measured in parallel, so only the lipid ratios could be compared.

## 5. Conclusions

A specific profile of serum PI species is associated with chronic HCV infection, whereas liver cirrhosis has a comparatively low effect on the serum PI profile. This study showed that serum PI species are not suitable biomarkers for monitoring the severity of liver disease or for detecting liver cirrhosis non-invasively in patients with HCV.

## Figures and Tables

**Table 1:** Median, minimum, and maximum serum PI levels of patients with HCV before therapy. Data of patients with and without liver cirrhosis are given.

PI [nmol/mL]	HCV Patients Without Cirrhosis			HCV Patients with Cirrhosis			*p*-Value
	Median	Minimum	Maximum	Median	Minimum	Maximum	
34:1	5.39	1.53	14.89	6.93	2.39	12.18	<0.05
34:2	3.66	1.54	9.11	4.50	1.58	6.92	Not significant
36:1	5.07	1.90	12.04	7.20	2.17	19.19	<0.05
36:2	13.00	7.50	31.22	13.94	5.28	28.53	Not significant
36:3	2.66	1.38	5.74	3.51	1.39	6.27	<0.01
36:4	3.96	1.30	9.44	3.85	1.51	7.07	Not significant
38:2	0.51	0.00	1.37	0.61	0.21	1.34	Not significant
38:3	6.35	3.20	15.65	5.79	2.28	13.95	Not significant
38:4	36.39	19.39	62.87	28.96	11.82	52.36	<0.001
38:5	2.48	1.30	6.68	2.69	0.92	4.79	Not significant
38:6	0.93	0.22	2.62	0.86	0.39	2.03	Not significant
40:4	0.68	0.32	1.16	0.55	0.25	1.23	Not significant
40:5	1.83	0.59	3.27	1.39	0.65	2.92	<0.05
40:6	2.66	0.45	7.60	1.88	0.93	4.71	<0.01
Total PI	87.12	52.25	139.91	79.82	33.28	138.47	Not significant

**Table 2:** Laboratory parameters of the patients before and after antiviral therapy excluding patients with liver cirrhosis. The median, minimum and maximum values are shown in the table (alanine aminotransferase (ALT), aspartate aminotransferase (AST), body mass index (BMI), C-reactive protein (CRP), high-density lipoprotein (HDL), international normalized ratio (INR), low-density lipoprotein (LDL), model of end-stage liver disease (MELD), not significant (ns)).

Laboratory Parameter	Baseline (138 Patients)	12 Weeks of Therapy (136 Patients)	*p*-Value
Age years	51 (24–82)	51 (24–82)	ns
Female/Male	58/80	58/78	ns
BMI kg/m^2^	25.5 (17.6–40.4)	25.6 (17.6–40.4)	ns
MELD Score	7 (6–21)	7 (6–20)	ns
Ferritin ng/mL	136 (6–2309)	90 (3–620)	<0.05
ALT U/L	71 (19–305)	26 (6–388)	<0.001
AST U/L	44 (14–1230)	19 (6–836)	<0.001
Bilirubin mg/dL	0.6 (0–1.7)	0.5 (0.2–1.8)	ns
INR	1.0 (0.8–1.4)	1.0 (0.9–1.4)	ns
Creatinine mg/dL	0.8 (0.5–14.0)	0.8 (0.5–14.7)	ns
Platelets n/nL	216 (49–402)	224 (45–407)	ns
Leukocytes n/L	6.8 (2.2–72.4)	7.2 (2.6–62.9)	ns
CRP mg/L	2.9 (2.8–55.0)	2.9 (2.9–20.3)	ns
Procalcitonin ng/mL	0.06 (0–10.07)	0.03 (0.01–0.60)	<0.05
Albumin g/L	39 (29–46)	40 (32–93)	ns
HDL mg/dL	53 (19–111)	50 (23–89)	ns
LDL mg/dL	100 (23–219)	128 (49–251)	<0.001

**Table 3:** Correlation of PI species with laboratory measures of liver disease in patients without liver cirrhosis before therapy. The Spearman correlation coefficients and the *p*-values, which were corrected for 14 comparisons, are listed (alanine aminotransferase (ALT), aspartate aminotransferase (AST), high-density lipoprotein (HDL), low-density lipoprotein (LDL), model of end-stage liver disease (MELD)).

PI [nmol/mL]	MELD Score	ALT U/L	AST U/L	LDL mg/dL	HDL mg/dL	Leukocytes n/L
34:1	0.004	0.116	0.242	0.131	−0.021	−0.030
34:2	0.039	0.121	0.245	0.152	0.126	−0.133
36:1	−0.065	0.031	0.115	0.077	0.070	−0.064
36:2	−0.047	0.017	0.140	0.040	0.278 *^p^*^=0.020^	−0.205
36:3	0.046	0.017	0.208	0.009	0.206	−0.204
36:4	−0.010	0.118	0.223	0.206	0.023	−0.164
38:2	−0.060	0.001	0.152	0.176	0.177	−0.202
38:3	−0.019	0.000	0.134	0.208	0.184	−0.104
38:4	−0.152	−0.077	0.025	0.293 *^p^*^=0.011^	0.224	−0.213
38:5	0.047	0.023	0.215	0.082	0.253	−0.268 *^p^*^=0.021^
38:6	0.134	0.243	0.407 *^p^*^<0.001^	0.118	0.079	−0.182
40:4	0.002	0.097	0.236	0.260 *^p^*^=0.041^	−0.016	−0.090
40:5	0.030	0.114	0.258 *^p^*^=0.031^	0.177	0.231	−0.096
40:6	0.070	0.183	0.357 *^p^*^<0.001^	0.131	0.112	−0.109
Total PI	−0.058	0.023	0.187	0.233	0.230	−0.236

**Table 4:** Median PI species concentration at the end of treatment/median PI concentration before treatment in % of patients with and without liver cirrhosis.

PI [nmol/mL]	% Change in Non-Cirrhosis Patients	% Change in Cirrhosis Patients
34:1	93.19	98.15
34:2	98.41	102.67
36:1	100.12	85.94
36:2	93.83	94.04
36:3	119.53	113.52
36:4	121.91	116.78
38:2	109.34	101.31
38:3	121.95	115.90
38:4	116.31	104.18
38:5	125.56	121.97
38:6	91.79	112.94
40:4	94.98	104.62
40:5	81.78	101.40
40:6	83.79	97.18
Total PI	106.61	108.16

**Table 5:** Correlation of PI species with HDL and LDL in patients without liver cirrhosis at therapy end. The Spearman correlation coefficients and the *p*-values are listed.

PI [nmol/mL]	LDL mg/dL	HDL mg/dL
34:1	0.194	0.061
34:2	0.218	−0.013
36:1	0.254	0.132
36:2	0.227	0.114
36:3	0.251	0.153
36:4	0.318 *^p^*^=0.003^	−0.068
38:2	0.197	0.133
38:3	0.442 ^*p*<0.001^	0.138
38:4	0.518 ^*p*<0.001^	0.031
38:5	0.239	0.054
38:6	0.094	−0.023
40:4	0.362 ^*p*<0.001^	0.080
40:5	0.357 ^*p*<0.001^	0.068
40:6	0.145	0.068
Total PI	0.452 *^p^*^<0.001^	0.100

## Data Availability

The original contributions presented in this study are included in the article/supplementary material. Further inquiries can be directed to the corresponding author.

## Supplementary Materials

The following supporting information can be downloaded at: https://www.mdpi.com/article/10.3390/biomedicines13112720/s1, Table S1. Checklist PI. Figure S1. PI species/total PI (%) in the serum of patients without liver cirrhosis during therapy. Table S2: PI species levels of patients with and without liver cirrhosis according to the fibrosis-4 (FIB-4) score.

## Abbreviations

The following abbreviations are used in this manuscript:

ALT	alanine aminotransferase
AST	aspartate aminotransferase
BMI	body mass index
DAA	direct-acting antiviral
FIB-4	fibrosis-4
HCV	hepatitis C virus
HDL	high-density lipoprotein
INR	international normalized ratio
LDL	low-density lipoprotein
MELD	model of end-stage liver disease
PI	Phosphatidylinositol

## References

[1] Casiraghi M.A., De Paschale M., Romano L., Biffi R., Assi A., Binelli G., Zanetti A.R. (2004). Long-term outcome (35 years) of hepatitis C after acquisition of infection through mini transfusions of blood given at birth. Hepatology.

[2] Pawlotsky J.M., Negro F., Aghemo A., Berenguer M., Dalgard O., Dusheiko G., Marra F., Puoti M., Wedemeyer H., European Association for the Study of the Liver (2020). EASL recommendations on treatment of hepatitis C: Final update of the series. J. Hepatol..

[3] Rockey D.C., Friedman S.L. (2021). Fibrosis Regression After Eradication of Hepatitis C Virus: From Bench to Bedside. Gastroenterology.

[4] Peschel G., Grimm J., Buechler C., Gunckel M., Pollinger K., Aschenbrenner E., Kammerer S., Jung E.M., Haimerl M., Werner J. (2021). Liver stiffness assessed by shear-wave elastography declines in parallel with immunoregulatory proteins in patients with chronic HCV infection during DAA therapy. Clin. Hemorheol. Microcirc..

[5] Broquetas T., Herruzo-Pino P., Marino Z., Naranjo D., Vergara M., Morillas R.M., Forns X., Carrion J.A. (2021). Elastography is unable to exclude cirrhosis after sustained virological response in HCV-infected patients with advanced chronic liver disease. Liver Int..

[6] Grassi G., Di Caprio G., Fimia G.M., Ippolito G., Tripodi M., Alonzi T. (2016). Hepatitis C virus relies on lipoproteins for its life cycle. World J. Gastroenterol..

[7] Sidorkiewicz M. (2021). Hepatitis C Virus Uses Host Lipids to Its Own Advantage. Metabolites.

[8] Endo D., Satoh K., Shimada N., Hokari A., Aizawa Y. (2017). Impact of interferon-free antivirus therapy on lipid profiles in patients with chronic hepatitis C genotype 1b. World J. Gastroenterol..

[9] Hashimoto S., Yatsuhashi H., Abiru S., Yamasaki K., Komori A., Nagaoka S., Saeki A., Uchida S., Bekki S., Kugiyama Y. (2016). Rapid Increase in Serum Low-Density Lipoprotein Cholesterol Concentration during Hepatitis C Interferon-Free Treatment. PLoS ONE.

[10] Peschel G., Grimm J., Gulow K., Muller M., Buechler C., Weigand K. (2020). Chemerin Is a Valuable Biomarker in Patients with HCV Infection and Correlates with Liver Injury. Diagnostics.

[11] Villani R., Di Cosimo F., Romano A.D., Sangineto M., Serviddio G. (2021). Serum lipid profile in HCV patients treated with direct-acting antivirals: A systematic review and meta-analysis. Sci. Rep..

[12] Peschel G., Krautbauer S., Weigand K., Grimm J., Höring M., Liebisch G., Müller M., Buechler C. (2024). Rising Lysophosphatidylcholine Levels Post-Hepatitis C Clearance. Int. J. Mol. Sci..

[13] Wiesner P., Leidl K., Boettcher A., Schmitz G., Liebisch G. (2009). Lipid profiling of FPLC-separated lipoprotein fractions by electrospray ionization tandem mass spectrometry. J. Lipid Res..

[14] Blunsom N.J., Cockcroft S. (2020). Phosphatidylinositol synthesis at the endoplasmic reticulum. Biochim. Biophys. Acta Mol. Cell Biol. Lipids.

[15] D’Souza K., Epand R.M. (2014). Enrichment of phosphatidylinositols with specific acyl chains. Biochim. Biophys. Acta.

[16] Burgess J.W., Boucher J., Neville T.A., Rouillard P., Stamler C., Zachariah S., Sparks D.L. (2003). Phosphatidylinositol promotes cholesterol transport and excretion. J. Lipid Res..

[17] Stamler C.J., Breznan D., Neville T.A., Viau F.J., Camlioglu E., Sparks D.L. (2000). Phosphatidylinositol promotes cholesterol transport in vivo. J. Lipid Res..

[18] Badawi A., Di Giuseppe G., Arora P. (2018). Cardiovascular disease risk in patients with hepatitis C infection: Results from two general population health surveys in Canada and the United States (2007–2017). PLoS ONE.

[19] Luukkonen P.K., Zhou Y., Hyotylainen T., Leivonen M., Arola J., Orho-Melander M., Oresic M., Yki-Jarvinen H. (2016). The MBOAT7 variant rs641738 alters hepatic phosphatidylinositols and increases severity of non-alcoholic fatty liver disease in humans. J. Hepatol..

[20] Youssef S., El Razek Abbas E., Aly Y., Seif S. (2020). The Correlation between Single Nucleotide Polymorphism of MBOAT7 and PNPLA3 Genes to the Degree of Hepatic Fibrosis in HCV Patients: An Experience from Egypt. J. Biosci. Appl. Res..

[21] Buch S., Stickel F., Trepo E., Way M., Herrmann A., Nischalke H.D., Brosch M., Rosendahl J., Berg T., Ridinger M. (2015). A genome-wide association study confirms PNPLA3 and identifies TM6SF2 and MBOAT7 as risk loci for alcohol-related cirrhosis. Nat. Genet..

[22] Meroni M., Longo M., Fracanzani A.L., Dongiovanni P. (2020). MBOAT7 down-regulation by genetic and environmental factors predisposes to MAFLD. EBioMedicine.

[23] Mancina R.M., Dongiovanni P., Petta S., Pingitore P., Meroni M., Rametta R., Boren J., Montalcini T., Pujia A., Wiklund O. (2016). The MBOAT7-TMC4 Variant rs641738 Increases Risk of Nonalcoholic Fatty Liver Disease in Individuals of European Descent. Gastroenterology.

[24] Thabet K., Asimakopoulos A., Shojaei M., Romero-Gomez M., Mangia A., Irving W.L., Berg T., Dore G.J., Gronbaek H., Sheridan D. (2016). MBOAT7 rs641738 increases risk of liver inflammation and transition to fibrosis in chronic hepatitis C. Nat. Commun..

[25] Ezzikouri S., Elfihry R., Chihab H., Elmessaoudi-Idrissi M., Zaidane I., Jadid F.Z., Karami A., Tahiri M., Elhabazi A., Kabine M. (2018). Effect of MBOAT7 variant on hepatitis B and C infections in Moroccan patients. Sci. Rep..

[26] Ma D.W., Arendt B.M., Hillyer L.M., Fung S.K., McGilvray I., Guindi M., Allard J.P. (2016). Plasma phospholipids and fatty acid composition differ between liver biopsy-proven nonalcoholic fatty liver disease and healthy subjects. Nutr. Diabetes.

[27] Tiwari-Heckler S., Gan-Schreier H., Stremmel W., Chamulitrat W., Pathil A. (2018). Circulating Phospholipid Patterns in NAFLD Patients Associated with a Combination of Metabolic Risk Factors. Nutrients.

[28] Buechler C., Aslanidis C. (2020). Role of lipids in pathophysiology, diagnosis and therapy of hepatocellular carcinoma. Biochim. Biophys. Acta Mol. Cell Biol. Lipids.

[29] Ten Hove M., Pater L., Storm G., Weiskirchen S., Weiskirchen R., Lammers T., Bansal R. (2020). The hepatic lipidome: From basic science to clinical translation. Adv. Drug Deliv. Rev..

[30] McPhail M.J.W., Shawcross D.L., Lewis M.R., Coltart I., Want E.J., Antoniades C.G., Veselkov K., Triantafyllou E., Patel V., Pop O. (2016). Multivariate metabotyping of plasma predicts survival in patients with decompensated cirrhosis. J. Hepatol..

[31] Kunz F., Kosin D. (1970). Plasma phospholipids in cirrhosis of liver and fatty liver. Clin. Chim. Acta.

[32] Kiser J.J., Burton J.R., Anderson P.L., Everson G.T. (2012). Review and management of drug interactions with boceprevir and telaprevir. Hepatology.

[33] Yen Y.H., Kuo F.Y., Chen C.H., Hu T.H., Lu S.N., Wang J.H., Hung C.H. (2019). Ultrasound is highly specific in diagnosing compensated cirrhosis in chronic hepatitis C patients in real world clinical practice. Medicine.

[34] McPherson S., Hardy T., Dufour J.F., Petta S., Romero-Gomez M., Allison M., Oliveira C.P., Francque S., Van Gaal L., Schattenberg J.M. (2017). Age as a Confounding Factor for the Accurate Non-Invasive Diagnosis of Advanced NAFLD Fibrosis. Am. J. Gastroenterol..

[35] Bligh E.G., Dyer W.J. (1959). A rapid method of total lipid extraction and purification. Can. J. Biochem. Physiol..

[36] Liebisch G., Lieser B., Rathenberg J., Drobnik W., Schmitz G. (2004). High-throughput quantification of phosphatidylcholine and sphingomyelin by electrospray ionization tandem mass spectrometry coupled with isotope correction algorithm. Biochim. Biophys. Acta.

[37] Leidl K., Liebisch G., Richter D., Schmitz G. (2008). Mass spectrometric analysis of lipid species of human circulating blood cells. Biochim. Biophys. Acta.

[38] Kopczynski D., Ejsing C.S., McDonald J.G., Bamba T., Baker E.S., Bertrand-Michel J., Brugger B., Coman C., Ellis S.R., Garrett T.J. (2024). The lipidomics reporting checklist a framework for transparency of lipidomic experiments and repurposing resource data. J. Lipid Res..

[39] Liebisch G., Fahy E., Aoki J., Dennis E.A., Durand T., Ejsing C.S., Fedorova M., Feussner I., Griffiths W.J., Kofeler H. (2020). Update on LIPID MAPS classification, nomenclature, and shorthand notation for MS-derived lipid structures. J. Lipid Res..

[40] Hengst J., Falk C.S., Schlaphoff V., Deterding K., Manns M.P., Cornberg M., Wedemeyer H. (2016). Direct-Acting Antiviral-Induced Hepatitis C Virus Clearance Does Not Completely Restore the Altered Cytokine and Chemokine Milieu in Patients With Chronic Hepatitis C. J. Infect. Dis..

[41] Mascia C., Vita S., Zuccala P., Marocco R., Tieghi T., Savinelli S., Rossi R., Iannetta M., Pozzetto I., Furlan C. (2017). Changes in inflammatory biomarkers in HCV-infected patients undergoing direct acting antiviral-containing regimens with or without interferon. PLoS ONE.

[42] Mauss S., Berger F., Wehmeyer M.H., Ingiliz P., Hueppe D., Lutz T., Simon K.G., Schewe K., Rockstroh J.K., Baumgarten A. (2017). Effect of antiviral therapy for HCV on lipid levels. Antivir. Ther..

[43] Sagnelli E., Sagnelli C., Russo A., Pisaturo M., Camaioni C., Astorri R., Coppola N. (2021). Impact of DAA-Based Regimens on HCV-Related Extra-Hepatic Damage: A Narrative Review. Adv. Exp. Med. Biol..

[44] Weigand K., Peschel G., Grimm J., Horing M., Krautbauer S., Liebisch G., Muller M., Buechler C. (2024). Serum Phosphatidylcholine Species 32:0 as a Biomarker for Liver Cirrhosis Pre- and Post-Hepatitis C Virus Clearance. Int. J. Mol. Sci..

[45] Vallet-Pichard A., Mallet V., Nalpas B., Verkarre V., Nalpas A., Dhalluin-Venier V., Fontaine H., Pol S. (2007). FIB-4: An inexpensive and accurate marker of fibrosis in HCV infection. Comparison with liver biopsy and fibrotest. Hepatology.

[46] Dzekova-Vidimliski P., Dzikova S., Selim G., Gelev S., Trajceska L., Pushevski V., Sikole A. (2013). Ultrasound predictors of compensated liver cirrhosis in hemodialysis patients with hepatitis C. Saudi J. Kidney Dis. Transpl..

[47] Horowitz J.M., Venkatesh S.K., Ehman R.L., Jhaveri K., Kamath P., Ohliger M.A., Samir A.E., Silva A.C., Taouli B., Torbenson M.S. (2017). Evaluation of hepatic fibrosis: A review from the society of abdominal radiology disease focus panel. Abdom. Radiol..

[48] Lambrecht J., Verhulst S., Mannaerts I., Reynaert H., van Grunsven L.A. (2018). Prospects in non-invasive assessment of liver fibrosis: Liquid biopsy as the future gold standard?. Biochim. Biophys. Acta Mol. Basis Dis..

[49] Bachofner J.A., Valli P.V., Kroger A., Bergamin I., Kunzler P., Baserga A., Braun D., Seifert B., Moncsek A., Fehr J. (2017). Direct antiviral agent treatment of chronic hepatitis C results in rapid regression of transient elastography and fibrosis markers fibrosis-4 score and aspartate aminotransferase-platelet ratio index. Liver Int..

[50] Bassani L., Fernandes S.A., Raimundo F.V., Harter D.L., Gonzalez M.C., Marroni C.A. (2015). Lipid profile of cirrhotic patients and its association with prognostic scores: A cross-sectional study. Arq. Gastroenterol..

[51] Barneda D., Cosulich S., Stephens L., Hawkins P. (2019). How is the acyl chain composition of phosphoinositides created and does it matter?. Biochem. Soc. Trans..

[52] Bone L.N., Dayam R.M., Lee M., Kono N., Fairn G.D., Arai H., Botelho R.J., Antonescu C.N. (2017). The acyltransferase LYCAT controls specific phosphoinositides and related membrane traffic. Mol. Biol. Cell..

[53] Milosevic I., Todorovic N., Filipovic A., Simic J., Markovic M., Stevanovic O., Malinic J., Katanic N., Mitrovic N., Nikolic N. (2023). HCV and HCC Tango-Deciphering the Intricate Dance of Disease: A Review Article. Int. J. Mol. Sci..

[54] Mita E., Hayashi N., Kanazawa Y., Hagiwara H., Ueda K., Kasahara A., Fusamoto H., Kamada T. (1994). Hepatitis C virus genotype and RNA titer in the progression of type C chronic liver disease. J. Hepatol..

[55] Ran X., Xu Y., Wang Y., Zeng C., Gong C., Wang N., Cai D. (2025). Genotype 3 is linked to worse liver disease progression in hepatitis C patients even after SVR following DAA therapy. Front. Cell. Infect. Microbiol..

[56] Cerda A., Bortolin R.H., Yoshinaga M.Y., Freitas R.C.C., Dagli-Hernandez C., Borges J.B., Oliveira V.F., Goncalves R.M., Faludi A.A., Bastos G.M. (2023). Lipidomic analysis identified potential predictive biomarkers of statin response in subjects with Familial hypercholesterolemia. Chem. Phys. Lipids.

[57] Zarezadeh M., Mahmoudinezhad M., Faghfouri A.H., Radkhah N., Jamali M., Jamilian P., Ghoreyshi Z., Ostadrahimi A. (2025). Serum phospholipids during aging: A comprehensive systematic review of cross-sectional and case-control studies. Health Promot. Perspect.

[58] West A.L., Michaelson L.V., Miles E.A., Haslam R.P., Lillycrop K.A., Georgescu R., Han L., Napier J.A., Calder P.C., Burdge G.C. (2021). Lipidomic Analysis of Plasma from Healthy Men and Women Shows Phospholipid Class and Molecular Species Differences between Sexes. Lipids.

